# Syntax-Sensitive Regions of the Posterior Inferior Frontal Gyrus and the Posterior Temporal Lobe Are Differentially Recruited by Production and Perception

**DOI:** 10.1093/texcom/tgaa029

**Published:** 2020-07-01

**Authors:** William Matchin, Emily Wood

**Affiliations:** Communication Sciences and Disorders, University of South Carolina, Columbia, SC 29208, USA

**Keywords:** inferior frontal gyrus, language-selective ROIs, posterior temporal lobe, production, syntax

## Abstract

Matchin and Hickok (2020) proposed that the left posterior inferior frontal gyrus (PIFG) and the left posterior temporal lobe (PTL) both play a role in syntactic processing, broadly construed, attributing distinct functions to these regions with respect to production and perception. Consistent with this hypothesis, functional dissociations between these regions have been demonstrated with respect to lesion–symptom mapping in aphasia. However, neuroimaging studies of syntactic comprehension typically show similar activations in these regions. In order to identify whether these regions show distinct activation patterns with respect to syntactic perception and production, we performed an fMRI study contrasting the subvocal articulation and perception of structured jabberwocky phrases (syntactic), sequences of real words (lexical), and sequences of pseudowords (phonological). We defined two sets of language-selective regions of interest (ROIs) in individual subjects for the PIFG and the PTL using the contrasts [syntactic > lexical] and [syntactic > phonological]. We found robust significant interactions of comprehension and production between these 2 regions at the syntactic level, for both sets of language-selective ROIs. This suggests a core difference in the function of these regions with respect to production and perception, consistent with the lesion literature.

## Introduction

Although sentences appear as linear sequences of words, they are combined into hierarchical structures that determine their semantic interpretation (Chomsky 1957; Heim and Kratzer 1998). During online processing, syntactic mechanisms group words incrementally into these hierarchical structures (Crocker 1996; Schneider 1999; Lombardo and Sturt 2002; Sturt and Lombardo 2005). Neuroimaging studies of syntactic comprehension, such as the contrast of structured phrases or sentences to unstructured word lists, have revealed increased activation in a variety of left hemisphere brain regions (for a meta-analysis, see Zaccarella et al. 2017). These activations appear to be selective for higher-level linguistic computations, as these areas, when defined in individual subjects, do not show increased activation for a variety of nonlinguistic tasks (Fedorenko et al. 2011). By contrast, there are spatially adjacent domain-general regions that appear to respond to a variety of nonlinguistic tasks (Fedorenko et al. 2012a; Fedorenko et al. 2013).

However, there is good evidence to suggest that syntactic processing is more specific to 2 of these regions: the posterior inferior frontal gyrus (PIFG, roughly Broca’s area, consisting of the *pars opercularis* and *pars triangularis* combined) and the posterior temporal lobe (PTL). For instance, the comparison of noncanonical to canonical sentence structures, that is, the comparison of less frequent, syntactically demanding structures with more common, less demanding sentence structures, has primarily revealed activation in these 2 areas (for a meta-analysis, see Meyer and Friederici 2016). These 2 regions also selectively show increased activation for jabberwocky sentences, structured sentences with content words replaced with pseudowords (greatly reducing conceptual-semantic content), relative to scrambled jabberwocky sentences (Pallier et al. 2011; Fedorenko et al. 2012b; Goucha and Friederici 2015; Matchin et al. 2017). Finally, they also show increased activation for verb phrases (with more complex syntactic organization) relative to lexically matched noun phrases (with less complex syntactic organization), whereas other language-responsive regions do not exhibit this difference (Matchin et al. 2019). This suggests that language-selective portions of the PIFG and PTL support syntactic processing, broadly construed, whereas other regions more likely reflect semantic processes (Binder 2017; Matchin and Hickok 2020; Pylkkänen 2020).

However, neuroimaging studies have yet to ascertain a clear distinction of function between the PIFG and the posterior temporal lobe. Individual neuroimaging studies of syntactic processing have sometimes reported isolated syntactic effects in the PIFG without corresponding posterior temporal lobe effects (Stromswold et al. 1996; Caplan et al. 2000; Goucha and Friederici 2015; Zaccarella and Friederici 2015), but other studies reveal that both regions reliably exhibit these effects (Ben-Shachar et al. 2003, 2004; Bornkessel et al. 2005; Rogalsky et al. 2008; Obleser et al. 2011; Pallier et al. 2011; Fedorenko et al. 2012b; Matchin et al. 2017). Therefore, from the perspective of neuroimaging, it is unclear what the functional dissociation of these regions is (if any) with respect to syntax.

While the neuroimaging literature does not provide clear evidence of a functional distinction between the PIFG and the PTL, lesion–symptom mapping analyses in aphasia have revealed distinct syntactic deficits following damage to these regions. Damage to PTL is associated with sentence comprehension and syntactic perception deficits, when confounding effects of working memory resources are accounted for (Dronkers et al. 2004; Wilson and Saygın 2004; Pillay et al. 2017; Rogalsky et al. 2018; Matchin and Hickok 2020). In addition, (Matchin et al. 2020) found that agrammatic production deficits (overall omission of functional elements and simplification of sentence structure) are associated with damage to the PIFG but not PTL, whereas paragrammatic production deficits (grammatical errors with no overall omission/simplification) are associated with damage to PTL but not the PIFG. Examples of agrammatic speech (1–3) and paragrammatic speech (4–6) (from Matchin et al. 2020) are shown below, illustrating the qualitatively distinct impairments in these syndromes:

(1) Cinderella one shoe (agrammatic)
(2) Two girls and boy bad (agrammatic)
(3) Cinderella all dressed and… slippers (agrammatic)
(4) …wanted to make a trick her (paragrammatic)
(5) …tooked her dress (paragrammatic)
(6) The queen and king is there (paragrammatic)

Such results support Matchin and Hickok (2020) that there is in fact a functional dissociation between these regions with respect to syntax, such that both areas are critically implicated in production, but only the PTL is critically implicated in perception. However, while there are numerous neuroimaging studies of syntactic perception, few studies have attempted to isolate morpho-syntactic aspects of production (Haller et al. 2005; Schönberger et al. 2014; Matchin and Hickok 2016) or directly compare production and comprehension of syntax within the same study (Menenti et al. 2011, 2012; Segaert et al. 2012, 2013). Two recent MEG studies found increased activation for phrase relative to list production in the anterior temporal lobe but not in the PIFG (Del Prato and Pylkkänen 2014; Pylkkänen et al. 2014); however, this experimental paradigm has been interpreted as reflecting conceptual composition rather than syntactic processes (Pylkkänen 2020). Thus, the paucity of studies that have investigated syntactic aspects of speech production may potentially account for the limited neuroimaging evidence for distinctions in syntactic processing between the PIFG and the PTL.

Extant theories of language in the brain do not discuss production–comprehension asymmetries but rather discuss higher-level linguistic functions such as hierarchical structure building (Friederici, 20 127), unification (Hagoort 2014), sequencing (Bornkessel-Schlesewsky and Schlesewsky 2013), morphological processes (Tyler and Marslen-Wilson 2008), and the processing of meaning (Fedorenko and Blank 2020). This is consistent with a long-standing assertion in linguistic theory regarding shared central computational resources between production and comprehension (Chomsky 1965; Jackendoff 2002; Momma and Phillips 2018). However, the existence of shared resources does not address the fact that production and comprehension have distinct computational demands. Some mechanisms may be tuned more for 1 task than for the other, even though they may each be involved in both tasks to some extent.

In the present study, we decided to test the hypothesis offered in Matchin and Hickok (2020), that the left PIFG and the PTL underlie syntactic processing asymmetrically with respect to perception and production, by assessing syntactic perception and production in the brain in the same fMRI study. We used simple linguistic materials consisting of sequences of two-word jabberwocky structures (e.g., “this pand these clopes”), and a “perceive and rehearse” paradigm used in previous studies to localize both speech production and perception (Buchsbaum et al. 2001; Hickok et al. 2003; Okada and Hickok 2009; Isenberg et al. 2012; Venezia et al. 2016). We expected that language-selective subregions of the PIFG and the PTL (identified in individual subjects) would exhibit increased activation for syntactically structured materials relative to unstructured word and nonword lists, consistent with previous findings. However, we hypothesized that production and perception would differentially recruit these regions: language-selective subregions of the PIFG would be preferentially recruited by production, and language-selective subregions of the PTL would be more equally driven by production and perception.

## Materials and Methods

### Subjects

We recruited 20 healthy, right-handed, native speakers of English with no history of neurological dysfunction (age 18–32, average 21.9). Subjects were paid $25 an hour for 2 hours of participation, for a total of $50 in total compensation. All subjects gave informed consent to participate, and all procedures were approved by the Institutional Review Board of the University of South Carolina.

### Stimuli

The experiment was comprised of a 3 × 3 design: 3 different tasks (*perceive + rest*, *perceive + rehearse*, *continuous perceive*) by 3 levels of content (phonological, syntactic, lexical). As we discuss in more detail later, for statistical comparisons, we used the contrast of *perceive + rehearse* > *perceive + rest* to define the effect of production, whereas continuous perceive > rest (or activation relative to the absence of a task) was used to define the effect of perception. This ensured that both production and perception involved 3 stimulus iterations.

#### Phonological Stimuli

We created the phonological materials by generating pseudowords for which no syntactic category was obvious. We created 16 total bisyllabic pseudowords, roughly distributed across different speech segments divided into 2 sets, with the constraint of syllable structure CV, a single onset consonant with an optional single code consonant. Each phonological stimulus consisted of a sequence of 2 pseudowords, 1 (initial position) drawn from Set 1, and the second (second position) drawn from Set 1. Set 1 (initial position) consisted of the following: *perwoth*, *nansow*, *ninyo*, *denferr*, *bulbom*, *nillex*, *seenig*, *tringess*. Set 2 (second position) consisted of the following: *lerris*, *foyrix*, *pobset*, *ganliff*, *demesh*, *garlay*, *susset*, *furgle*. Each pseudoword from the first set was paired once with all of the pseudowords from the second set, producing 64 unique two-pseudoword sequences (e.g., *perwoth lerris*), with the first word presented on top of the second word on the screen during the experiment. We then repeated these 64 sequences for use across the entire experiment, copying the set of 64 once and then randomly selecting an additional subset of 22/64 sequences for a total of 150 sequences. We then distributed these 150 sequences randomly to create 30 trials each for the 3 task conditions, using 1 stimulus for each perceive+rest trial, 1 stimulus for each perceive+rehearse trial, and 3 stimuli for each continuous perceive trial.

We operated under the assumption that pseudowords/nonwords would exist as phonological strings without syntactic or conceptual–semantic content. Thus, we assumed that it was critical to match phonological materials with the other conditions with respect to number of syllables (4 per stimulus) rather than number of words/pseudowords. We chose to present 2 bisyllabic pseudowords rather than 4 monosyllabic pseudowords to attempt to reduce the working memory burden of having to remember 4 distinct chunks in the phonological condition during perceive+rehearse trials (Cowan 2001).

#### Syntactic Stimuli

We created the syntactic materials by adapting the phonologically plausible pseudoword nouns created by Matchin et al. (2017) using the Wuggy software (Keuleers and Brysbaert 2010). This study created phonologically plausible pseudowords (nouns) preceded by real determiners to create phrases that preserved syntactic structure but greatly reducing conceptual content in order to investigate the neural bases of syntactic processing. They were designed to match the phonological plausibility of real nouns used within that study. We selected monosyllabic pseudoword nouns such that each jabberwocky phrase contained 2 syllables to match the phonological condition. The monosyllabic pseudowords had a syllable structure of (C)CVC(C), that is, minimally a CVC with an optional additional onset OR coda consonant. i.e., the 3 possible syllables were CVC, CCVC, and CVCC. The final set was as follows: *bleff*, *woon*, *pand*, *delk*, *sheeve*, *glit*, *lart*, *clope*. We then combined these pseudowords with a set of 8 determiners to create 64 unique phrases: the articles *a* and *the*, possessive pronouns *his* and *their*, demonstratives *this* and *those*, and the quantifiers *each* and *few*. In order to ensure variability in syntactic number features across stimuli, *a*, *their*, *this*, and *each* were combined with a singular noun (e.g., *a bleff*) while *the*, *his*, *those*, and *few* were combined with a plural noun (e.g., *the pands*). In order to create stimuli that matched the phonological condition in number of syllables, we combined 2 phrases together to form each individual stimulus, randomly assigned with the constraint that the 2 phrases not overlap in either the pseudoword or determiner and balanced to have equal numbers of each determiner in both the first and second phrases. This resulted in 64 two-phrase sequences (e.g., *these clopes this pand*), with the first phrase presented on top of the second phrase on the screen during the experiment. As with the phonological stimuli, we duplicated this set of 64 and added 22/64 randomly selected phrases to create a total of 150 sequences. We then distributed these 150 sequences randomly to create 30 trials each for the 3 task conditions, using 1 stimulus for each perceive+rest trial, 1 stimulus for each perceive+rehearse trial, and 3 stimuli for each continuous perceive trial.

#### Lexical Stimuli

We created the lexical materials by combining 2 semantically unrelated bisyllabic nouns each consisting of 2 syllables. From a set of 16 nouns, we divided them into 2 sets. Set 1 (initial position) consisted of the following: *hermit*, *ninja*, *pirate*, *poet*, *sheriff*, *mutant*, *glutton*, *hostage*. Set 2 (second position) consisted of the following: *dogma*, *vodka*, *garbage*, *pistol*, *organ*, *fortress*, *scandal*, *robot*. Each word from the first set was paired once with all of the words from the second set, producing 64 unique two-word sequences (e.g., *hermit dogma*), with the first word presented on top of the second word on the screen during the experiment. We then repeated these 64 sequences for use across the entire experiment, copying the set of 64 once and then randomly selecting an additional subset of 22/64 sequences for a total of 150 sequences. We then distributed these 150 sequences randomly to create 30 trials each for the 3 task conditions, using 1 stimulus for each perceive+rest trial, 1 stimulus for each perceive+rehearse trial, and 3 stimuli for each continuous perceive trial.

We matched the lexical stimuli with the syntactic stimuli by matching them on number of syllables (4 total syllables per stimulus) and number of real words (2 real words per stimulus). Another option would have been to match the lexical and syntactic conditions on the total number of words/pseudowords, for instance, by including 4 real words in the lexical condition. However, language-related brain region have been shown to respond to both lexicality contrasts (words > nonwords) and structural contrasts (phrases/sentences > lists) (Fedorenko et al. 2010; Matchin et al. 2017). Matching the lexical and syntactic conditions on total number of words/pseudowords, as opposed to number of real words, might eliminate any increased activity in the syntactic condition relative to the lexical condition due to syntactic structure, as the lexical condition would have a greater number of real words. Thus we decided to hold the number of real words constant between these conditions.

### Procedure

**Figure 1:**
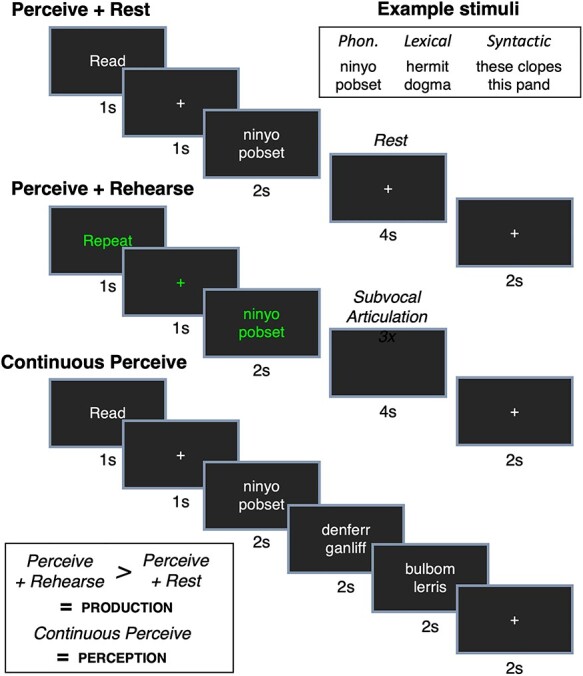
A schematic of stimulus design and presentation. See text for details. Phon. = phonological.

The experiment consisted of 2 phases: a training phase outside of the scanner during which subjects were exposed to all of the experimental conditions and the subvocal rehearsal task performed with overt speech production and the testing phase in which subjects performed the task covertly inside of the scanner. We asked subjects to perform the task overtly in the training phase in order to ensure that they could perform the task and so that they were able to receive feedback if necessary (e.g., reminding them to produce exactly 3 articulations), with the assumption that it would not be difficult to proceed with subvocal articulation in the testing phase. Stimuli were presented using Psychtoolbox (Brainard 1997; Kleiner et al. 2007). Task presentation was identical for the training and testing phases; the only difference was whether the subject articulated overtly (training phase) or imagined speaking (testing phase). All trials involved the presentation of a cue for 1 s: the word *read* (in white font) cueing the subject to comprehend the stimulus but not articulate, used for both the perceive+rest and continuous perceive conditions, or the word *repeat* (in green font), used for the perceive+repeat condition, cueing the subject to comprehend the stimulus and then repeat it 3 times during the delay phase. After the cue, a fixation cross was presented for 1 s in the same color font as the cue. Following this, a written speech stimulus was presented for 2 s. In the continuous perceive condition, 2 additional speech stimuli were presented for 2 s each, followed by a white fixation cross for 2 s before the next trial. In the perceive+rest condition, a white fixation cross was presented for 6 s. In the perceive+repeat condition, the screen was blank for 4 s during which the subject was trained to repeat the speech stimulus 3 times, followed by a white fixation cross for 2 s before the next trial. A schematic of stimulus presentation is shown in Figure 1.

#### Training Phase

During the training phase, subjects practiced all of the conditions (in both production and perception) that were presented in the final experiment, with a particular focus on ensuring that subjects could perform the subvocal rehearsal task. The experiment was first explained and modeled to the subject by the experimenter. Subjects were told that when the word “repeat” appeared in green font (the perceive+rehearse condition) to produce the presented speech stimulus 3 times during the delay period between fixation crosses, during which no nothing appeared on the screen. For the perceive+rest and continuous perceive conditions, the subjects were told that when the word ``read'' appeared in white font that they should read and comprehend the speech stimuli presented on screen but that they should not produce anything. They were instructed to perform the task out loud during the training phase, but that they would only imagine speaking during the testing phase inside the scanner.

Following this, subjects performed the training phase task in 2 short runs consisting of 24 trials each: 6 perceive+rest, 6 perceive+repeat, 6 continuous perceive, and 6 rest trials consisting of fixation only. Practice trials were randomly selected from the set of created stimuli and presented in random order, such that all conditions were balanced (i.e., 9 experimental conditions with 2 trials per condition). Random order was manually rearranged so that at least 2 non-rest trials intervened between rest trials and runs always ended with a rest trial. The 6 overt perceive+rehearse trials per run, with 3 overt utterances per trial, were recorded for later analysis (for 2 subjects, auditory recordings were unavailable due to equipment issues; therefore only 18 subjects’ data were analyzed).

#### Testing Phase

The experiment was divided into 9 runs of 40 trials each: 10 perceive+rest, 10 perceive+repeat, 10 continuous perceive, and 10 rest trials. The 9 conditions (phonological perceive+rest, phonological perceive+rehearse, phonological continuous perceive, lexical perceive+rest, lexical perceive+rehearse, lexical continuous perceive, syntactic perceive+rest, syntactic perceive+rehearse, syntactic continuous perceive) were maximally balanced across runs. For example, in 1 run, for the 10 perceive+rest trials, 3 were phonological, 3 syntactic, and 4 lexical. As in the practice run, at least 2 non-rest trials intervened between rest trials, and runs always ended on rest trials (to allow the BOLD response to return to baseline at the end of the run).

#### fMRI Data Collection and Analysis

Brain data were obtained in a Siemens PRISMA 3 T scanner (Siemens Medical Systems) using a 20-channel head coil. After the subjects were installed in the scanner, preliminary scans were obtained in order to localize the subject’s brain and adjust shim coils for magnetic field homogeneity. The subject was reminded not to produce any speech out loud but only to subvocally rehearse in the perceive+repeat trials. Following this, the subject performed 4 experimental runs, followed by a high-resolution T1 anatomical scan, followed by the last 5 runs. Each run lasted approximately 6 min, and some subjects occasionally took a 1 min break in-between runs. Following the last run, the subject was removed from the scanner, debriefed, and paid for their participation.

The high-resolution T1-weighted anatomical image was collected in the axial plane (voxel dimension: 1 mm isotropic) using an MP-RAGE sequence (256 × 256 matrix size, 9 degree flip angle). A total of 2880 T2^*^-weighted EPI volumes were collected over 9 runs of 320 volumes apiece. Each volume consisted of 68 slices in ascending, interleaved order without gap (TR = 1260 ms, TE = 32 ms, flip angle = 45°, in-plane resolution = 2.5 × 2.5 mm, slice thickness = 2.5 mm with no gap). The first 4 volumes of each run (dummy volumes) were discarded automatically by the scanner to control for T1 saturation effects. Data were reconstructed using MRIcroGL (https://www.nitrc.org/projects/mricrogl). Slice-timing correction, motion correction, warping to MNI space, spatial smoothing, and conversion to percent signal change values were performed using AFNI software (Cox 1996) http://afni.nimh.nih.gov/afni). Motion correction was achieved by using a 6-parameter rigid- body transformation, with each functional volume in each run first aligned to a single volume in that run. Functional volumes were aligned to the anatomical image, aligned to MNI space, and resampled to 3 mm isotropic. Functional images were spatially smoothed using a Gaussian kernel of 6 mm FWHM.

First-level (individual subject) analysis was performed for each subject using AFNI’s 3dDeconvolve function. The regression equation identified parameter estimates that best explained variability in the data, using a canonical hemodynamic response function convolved with the timing of stimulus presentation for each condition. We included a regressor for each of the 9 conditions (phonological perceive+rest, phonological perceive+rehearse, phonological continuous perceive, lexical perceive+rest, lexical perceive+rehearse, lexical continuous perceive, syntactic perceive+rest, syntactic perceive+rehearse, syntactic continuous perceive), modeling the duration between the onset of the speech stimulus until the final fixation cross (6 s). We added a 2 s regressor for the cues (read, repeat) that preceded each speech stimulus. Finally, we included the 6 motion parameters as regressors of no interest. We then performed first-level contrasts to identify the effect of production (perceive+rehearse > perceive+rest) for each level of content (phonological, lexical, syntactic) within each subject. The effect of perception for each level of content was defined as the continuous perceive condition > rest (activation relative to scanning periods without any task).

#### Functional ROI Definition Procedure

We defined subject-specific functional ROIs within broader anatomical search spaces (Fedorenko et al. 2010; Rogalsky et al. 2015; Matchin et al. 2019). We first defined 2 localizer contrasts, orthogonal to our effects of interest, to identify language-selective subregions in individual subjects: all syntactic conditions (perceive+rest, perceive+rehearse, continuous perceive) compared to all lexical conditions, [syntactic > lexical] and all syntactic conditions compared to all phonological conditions, [syntactic > phonological]. We created anatomical search spaces by combining ROIs within the Johns Hopkins University atlas: the PIFG (inferior frontal gyrus, pars triangularis and pars opercularis) and the PTL (posterior superior temporal gyrus and middle temporal gyrus). We then intersected the subject-specific contrast maps thresholded at *P* < 0.005 with the anatomical search spaces to result in 4 individual ROIs for each subject: PIFG [syntactic > lexical], PIFG [syntactic > phonological], PTL [syntactic > lexical], and PTL [syntactic > phonological].

We used the syntactic > phonological contrast in order to localize language-response ROIs most similar to the ROI definition procedure of Fedorenko et al. (2010). However, given our interest in syntax, we also created ROIs similar to previous experiments investigating syntax in the brain by comparing sentences to real word lists (Rogalsky and Hickok 2009; Zaccarella et al. 2017). We therefore included both sets of ROIs such that we would ensure that we captured the relevant language-responsive subregions of the PIFG.

We then analyzed orthogonal functional dissociations within these regions at the group level, averaging the *t*-statistic for each condition across the voxels included within the subject-specific ROIs. We performed 6 separate 2 × 2 ANOVAs, separately for each linguistic level of content (phonological, lexical, and syntactic) and each Region defined by the localizer contrasts ([syntactic > lexical], [syntactic > phonological]). We analyzed the main effects of Task (perception vs. production), Region (PIFG, PTL), and their interaction.

Our whole-brain analyses were used to show the broader patterns of activation associated with our experimental manipulations. We created overlap maps to identify regions that showed increased activation for [syntactic > lexical] and [syntactic > phonological], separately for production and perception, using a voxel-wise threshold of *P* < 0.001, cluster size 40 voxels, which resulted in reported analyses passing an FDR correction for multiple comparisons at q < 0.05. In Supplementary Materials, we show whole-brain activations for all of the 6 individual effects across the 2 tasks (perception, production) × 3 linguistics levels (phonological, lexical, syntactic) design using these same statistical thresholds and FDR correction.

## Results

### Behavioral

**Figure 2:**
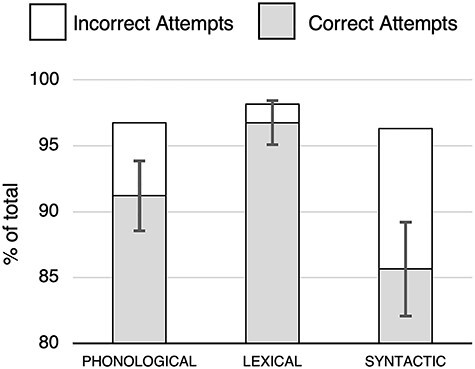
Behavioral data from the training phase. Data are shown as a percentage of the total possible number of utterances for each of the 3 levels of content (phonological, lexical, syntactic) during the perceive+rehearse task. Statistical analyses were performed on the proportion of accurate/attempted utterances. Error bars reflect standard error of the mean on the number of correctly attempted utterances. Note: in order to better display differences among conditions, the y-axis begins at 80% and not 0%.

Subjects performed well overall on attempting and accurately producing the presented speech stimuli during the training period prior to scanning (Fig. 2): 94% of attempted productions were accurate in the phonological condition, 88% of attempted productions were accurate in the syntactic condition, and 98% of attempted productions were accurate in the lexical condition. Paired samples *t*-tests revealed significantly better accuracy for phonological read+repeat relative to syntactic read+repeat, *t*(1,17) = 2.787, *P* = 0.013; significantly better accuracy for lexical read+repeat relative to syntactic read+repeat, *t*(1,17) = 4.136, *P* = 0.0006904; and significantly better accuracy for lexical read+repeat relative to phonological read+repeat, *t*(1,17) = 4.526, *P* = 0.0002986.

### fMRI—Whole-Brain Analyses

**Figure 3:**
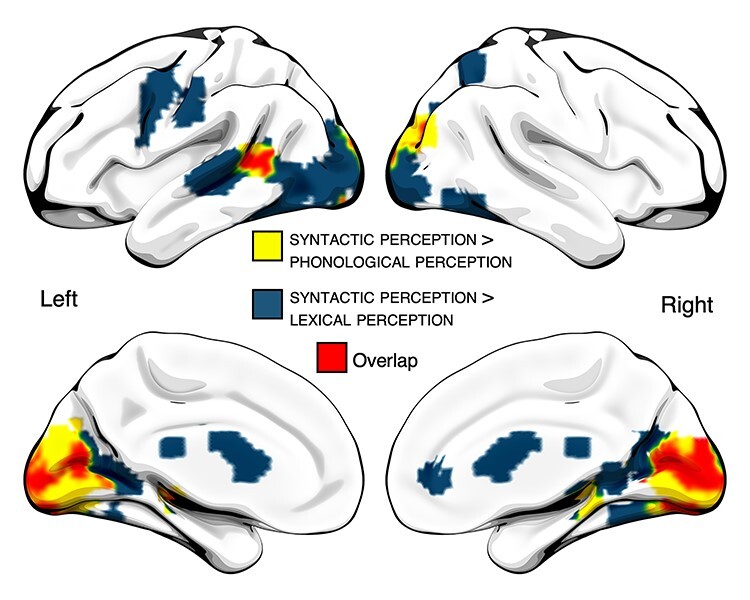
Conjunction (overlap) analysis of [syntactic > lexical] and [syntactic > phonological] in perception at the whole-brain level.

At the whole-brain level, no significant clusters emerged using for syntactic effects in production, the [syntactic > lexical] and [syntactic > phonological] contrasts. Significant clusters for syntactic effects in perception are shown in Figure 3. The [syntactic > lexical] contrast revealed activation in a broad set of regions, including left anterior precentral gyrus extending into the posterior inferior frontal gyrus; superior precentral gyrus; left pSTS/MTG; bilateral posterior ventral occipitotemporal cortex, bilateral dorsal occipital/parietal lobe, and bilateral calcarine sulcus (Fig. 3, dark blue). The [syntactic > phonological] contrast activated essentially a subset of these regions, including pSTS/MTG, bilateral calcarine sulcus, and dorsal occipital/parietal lobe (Fig. 3, yellow). Overlap between these effects was observed in left pSTS/MTG, bilateral calcarine sulcus, bilateral thalamus, and bilateral dorsal occipital/parietal lobe (Fig. 3, red). Center of mass coordinates for these effects is listed in Table 1.

**Table 1:** Center of mass coordinates, reported in MNI space, for clusters of increased activation for [syntactic > phonological] and [syntactic > lexical] in perception in the whole-brain conjunction/overlap analysis

Region	Size	X	Y	Z
Syntactic perception > phonological perception				
Bilateral calcarine sulcus	61 884 mm^3^	1	--84	4
Left pSTS/MTG	4293 mm^3^	--54	--49	9
Right thalamus	3375 mm^3^	15	--30	--9
Left thalamus	1566 mm^3^	--21	--24	--4
Syntactic perception > lexical perception				
Bilateral calcarine sulcus	76 464 mm^3^	--7	--75	1
Bilateral basal ganglia	16 929 mm^3^	--3	--25	17
Left precentral gyrus	9828 mm^3^	--49	--3	41
Right cerebellum	2052 mm^3^	25	--63	--53
Right superior parietal lobule	1566 mm^3^	26	--58	55
Right thalamus	1404 mm^3^	19	--26	--2
Left thalamus	1404 mm^3^	--22	--26	--4
Right anterior cingulate cortex	1107 mm^3^	12	39	4
“Overlap”				
Bilateral calcarine sulcus	7722 mm^3^	3	--84	2
Left pSTS/MTG	2538 mm^3^	--56	--49	9
Left thalamus	1080 mm^3^	--21	--25	--4
Right thalamus	621 mm^3^	20	--27	--3

### fMRI—ROI Analyses

**Figure 4:**
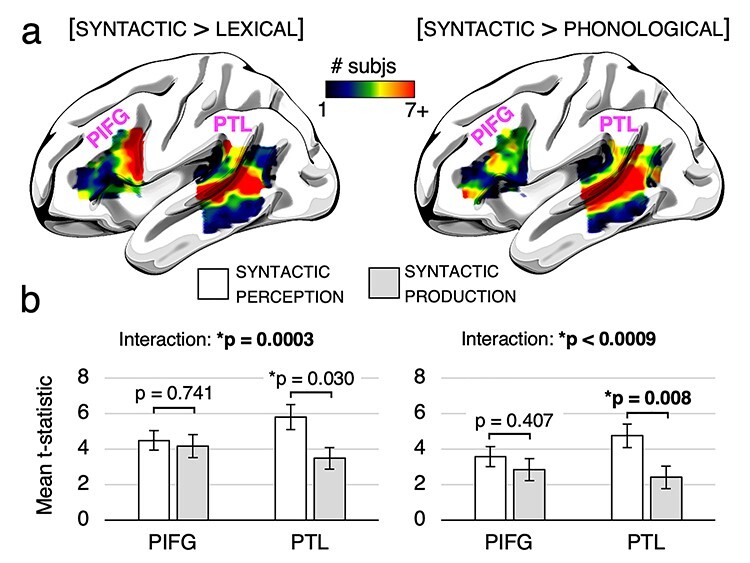
(*A*): Language-selective ROIs defined in individual subjects. Color indicates the number of subjects with overlapping significant voxels in each ROI. (*A*, left): ROIs defined by the localizer contrast [syntactic > lexical]. (*A*, right): ROIs defined by the localizer contrast [syntactic > phonological]. (*B*): Bar charts showing the average *t*-value within each ROI for syntactic perception and syntactic production. Bar charts on the left correspond to the functional ROIs defined using the [syntactic > lexical] contrast, and bar charts on the right correspond to the functional ROIs defined using the [syntactic > phonological] contrast. Error bars indicate standard error of the mean. PIFG = posterior inferior frontal gyrus, PTL = posterior temporal lobe. ^*^ indicates significance before multiple comparisons correction, and bolding indicates significance surviving a Bonferroni correction for multiple comparisons (4 pairwise simple main effects comparisons, *P* < 0.0125).

The 4 subject-specific functional ROIs are shown in Figure 4. For the localizer contrast [syntactic > lexical], maximum overlap in the PIFG (11 subjects) occurred in the pars opercularis, MNI peak coordinates [−47 10 22], and maximum overlap in the PTL (13 subjects) occurred in the superior temporal sulcus, MNI peak coordinates [−53, −41, 7]. For the localizer contrast [syntactic > phonological], 1 subject did not have significant voxels in the PIFG, so we omitted this subject’s data in all analyses for this functional localizer. Maximum overlap in the PIFG (7 subjects) occurred in 9 mostly noncontiguous voxels, with a general bias towards the pars triangularis (6 peak voxels) rather than pars opercularis (3 peak voxels), and maximum overlap in the PTL (14 subjects) occurred in the superior temporal sulcus, MNI peak coordinates [−58, −50, 8]. Overall, there was a high degree of correspondence between the 2 localizer contrasts in the PTL, but in the PIFG there was a noticeable distinction between the [syntactic > lexical] contrast (posterior) and the [syntactic > phonological] contrast (anterior).

The degree of overlap within these ROIs is less than reported Fedorenko et al. (2010). This study only reported overlap results for their strongest contrast, full natural sentences relative to nonword (pseudoword) lists. Out of 25 subjects (vs. 20 in the present study), they find maximum overlap in the PIFG of 14–15 subjects and maximum overlap in PTL of 18 subjects. Given that their sample was 20% bigger, a comparable degree of overlap would be 12 subjects in PIFG and 15 subjects in PTL. We found overlap of 7 subjects ([syntactic > phonological]) and 11 subjects ([syntactic > lexical]) in PIFG and overlap of 14 subjects ([syntactic > phonological]) and 13 subjects ([syntactic > lexical]) in the PTL. Thus, our numbers are comparable to theirs with respect to the PTL, comparable in the PIFG for 1 contrast, [syntactic > lexical], and 42% less for 1 contrast, [syntactic > phonological]. This last discrepancy is likely due to our more rigorous procedure for ROI definition (see Supplementary Materials for explanation).

The average *t*-statistic for each condition within each ROI is shown in Figure 4. For both ROI localizer contrasts, the PIFG had roughly equal activation for syntactic production and syntactic perception, and PTL had higher activation for syntactic perception relative to syntactic production. Table 2 contains the statistical results of the analyses performed within these ROIs. For the main effects of Task (syntactic perception vs. syntactic production) and Region (PIFG vs. PTL), our analyses revealed no significant differences, indicating that there were no overall differences in activation between the PIFG and the PTL or between syntactic perception and syntactic production. There was a highly significant interaction between Task and Region for both sets of ROIs, *P* < 0.001. Thus there was robust evidence for a production–perception asymmetry between language-selective subregions of the PIFG and PTL for syntactic processing. Simple main effects analyses revealed no significant difference between syntactic production and syntactic perception in the PIFG defined with either localizer, a marginally significant increase of activation for syntactic perception relative to syntactic production in the PTL defined via [syntactic > lexical] (not surviving a Bonferroni correction for multiple comparisons), and a significant increase of activation for syntactic perception relative to syntactic production in the PTL defined via [syntactic > phonological]. This suggests that the PIFG responded roughly equally for syntactic production, but that the PTL showed enhanced activity for syntactic perception.

**Table 2:** Statistical results of the ANOVAs comparing syntactic production and syntactic perception between the functionally defined, subject-specific ROIs in the PIFG and the posterior temporal lobe (PTL)

	[Syntactic > lexical] ROI		[Syntactic > phonological] ROI	
Region	*F*(1,19) = 0.524	*P* = 0.478	*F*(1,18) = 1.012	*P* = 0.328
Task	*F*(1,19) = 2.012	*P* = 0.172	*F*(1,18) = 3.674	*P* = 0.071
**Interaction**	***F*(1,19) = 19.524**	*P* = 0.0003^*^	***F*(1,18) = 15.945**	*P* = 0.0009^*^
Simple main effect of task for the PIFG	*F*(1,19) = 0.113	*P* = 0.741	*F*(1,18) = 0.722	*P* = 0.407
Simple main effect of task for the PTL	*F*(1,19) = 5.480	*P* = 0.030^*^	*F*(1,18) = 8.884	*P* = 0.008^*^

Notes: ^*^indicates significance before multiple comparisons correction, and bolding indicates significance surviving a Bonferroni correction for multiple comparisons (4 pairwise simple main effects, *P* < 0.0125).

## Discussion

The lack of strong differentiation between the activation profiles of the PIFG and PTL in neuroimaging studies of syntax, in contrast to readily identifiable distinctions in the lesion literature, poses a challenge to theories positing distinct syntactic contributions of these regions (Matchin and Hickok 2020). Consistent with previous studies, we identified syntax-sensitive subregions of both of these areas in individual subjects. However, we identified a clear asymmetry with respect to syntactic demands in production and perception: activation in the PIFG was driven more by the demands of production than PTL, and activation in the PTL was driven more by the demands of perception than the PIFG. This suggests that the syntactic functions of these regions are tuned to the distinct computational demands of production and perception, contrary to extant models of (morpho-)syntax in the brain that posit shared mechanisms (Tyler and Marslen-Wilson 2008; Bornkessel-Schlesewsky and Schlesewsky 2013; Hagoort 2014; Friederici 2017).

With respect to precise localization, our 2 individual subject localizer contrasts, [syntactic > lexical] and [syntactic > phonological], identified similar pSTS/MTG regions, both of which have been identified in previous studies of syntactic processing (e.g., Pallier et al. 2011; Matchin et al. 2017, 2019). At the group level, these contrasts overlapped in pSTS, suggesting a fairly robust role for this region in syntactic processing. However, our 2 localizer contrasts identified distinct subregions of the PIFG: the [syntactic > lexical] contrast highlighted pars opercularis, while the [syntactic > phonological] contrast highlighted pars triangularis. Additionally, at the group level, only the [syntactic > lexical] contrast found a significant effect in pars opercularis, with no significant effects for the [syntactic > phonological] contrast. While some studies have identified primary activation foci for syntactic processing in the pars triangularis (Pallier et al. 2011; Matchin et al. 2017, 2019), other studies have identified primary foci in the pars opercularis (e.g., Goucha and Friederici 2015; Zaccarella et al. 2017). The Matchin and Hickok (2020) model suggests that the key subregion for morpho-syntactic processing in the PIFG is the pars triangularis, based on the fact that phonological production demands (Buchsbaum et al. 2001; Hickok et al. 2003; Matchin et al. 2014), and phonological working memory demands in comprehension (Matchin et al. 2017, 2019) appear to drive activity in pars opercularis. We suggest that future research investigation the relationship between phonological processing and syntactic effects in pars opercularis be more thoroughly investigated.

One objection to the conclusion that syntax-sensitive regions of the PIFG are differentially driven by production/comprehension demands relative to the PTL is the hypothesis that this region contains distinct subregions, some of which are sensitive to higher-level syntax, some of which are sensitive to production, and that they are interdigitated, making it difficult to disentangle them. However, we identified our ROIs in individual subjects using language-selective localizer contrasts. Thus the interdigitated explanation is unlikely (although not impossible to rule out, if the interdigitation is finer than our voxel resolution), suggesting instead that language-selective regions of the PIFG reflect a distinct linguistic computation from that of the PTL.

While the PIFG has been implicated in production since the 1800s (Broca 1961), this role is not restricted to articulatory and/or phonological demands, at least in the pars triangularis. Recent electrocorticography studies of speech production have revealed that the PIFG is not active during speech articulation (Flinker et al. 2015) and is implicated in higher-level morphological processes (Moro et al. 2001; Sahin et al. 2009). Matchin and Hickok (2020) recently proposed that the pars triangularis area underlies a morpho-syntactic sequencing function, tied to the demands of production, whereas the posterior superior temporal sulcus/middle temporal gyrus (pSTS/MTG) is critically involved in hierarchical lexical-syntactic structuring, supporting both comprehension and production. The functional asymmetry that we observed in the present study is consistent with this proposal.

If the contribution of the PIFG to syntactic processing is mostly driven by the demands of production, why did we observe significant activation for syntactic processing in perception in this region? It is unlikely that this activation reflects working memory demands (cf. Rogalsky and Hickok 2011), as our stimuli involved maximally simple sequences of two-word phrases. We suggest, in agreement with other authors, that activation in Broca’s during perception in our task may reflect the prediction of upcoming material (Bonhage et al. 2015; Matchin et al. 2017; Matchin 2018; Rimmele et al. 2018). A role in top-down predictions is supported by the fact that lesions to IFG impair the rapid processing of syntactic violations (Jakuszeit et al. 2013) and, when sentence presentation is slowed, patients with IFG lesions show improved comprehension (Love et al. 2008). Thus some activation for syntactic perception is expected in this region, albeit asymmetrically with respect to the PTL, which appears to underlie core computations necessary for successful comprehension.

**Figure 5:**
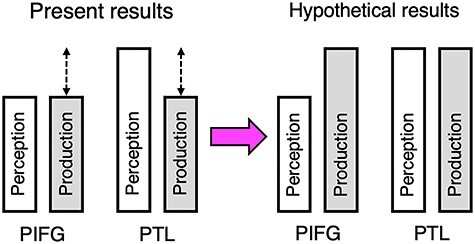
Left: schematization of the present results. Right: schematization of hypothetical results with increased morpho-syntactic production demands. White bars correspond to syntactic perception, and gray bars correspond to syntactic production.

We predicted a functional asymmetry between the PIFG and PTL such that PTL would be roughly equally activated by both perception and production of morpho-syntax, whereas PIFG would be more strongly recruited by production and perception (Fig. 5, right). However, we observed roughly equal activation for perception and production in PIFG and greater activation for perception relative to production in the PTL (Fig. 5, left). While this is broadly consistent with the functional asymmetry between perception and production proposed in Matchin and Hickok (2020), the lesion data suggest that the PTL is involved in both perception and production of syntax and a role for the PIFG restricted primarily to syntactic production. However, it may be that in our study, the articulatory rehearsal paradigm we used in the present study did not force subjects to always generate morpho-syntactic representations. Some subjects on some trials may have converted the syntactic representation to a phonological one and rehearsed a phonological, rather than morpho-syntactic, sequence. By contrast, the demands of natural sentence production require generating variable morpho-syntactic sequences. Future research using this paradigm should ensure that speech sequences cannot be rehearsed purely in a phonological code, but rather a task should be implemented that requires subjects to recode the morpho-syntactic structure of the utterance. We would predict that under such circumstances, activation in the PTL will be equivalent for perception and production, and there will be increased activation for production relative to perception in the PIFG (Fig. 5, right).

Another potential concern is that we did not attempt to match our production and perception conditions for overall difficulty. We note that we were not searching for a main effect of task but rather a task x region interaction effect. Thus any differences in terms of overall linguistic complexity between production and comprehension could not explain the task x region interaction. It could in theory be the case that the PIFG area was more selectively recruited by executive function resources than the PTL and if this was confound in our task, thereby explaining our data. However, 1 set of ROIs (defined with [syntactic > phonological] was defined in a very similar fashion as Fedorenko and colleagues have advocated, comparing structured linguistic materials to sequences of nonwords. Many studies by this group have shown that while domain-general task difficulty modulates brain activity in other regions, domain-general task difficulty does not modulate the response within these language-selective subregions (for a review, see Fedorenko and Blank 2020).

Finally, although our materials were matched for number of syllables, our whole-brain analyses revealed effects in the occipital lobe that were likely due to differences in the visual display among the conditions. Future research using a similar experimental design should explore other modalities of presentation, such as auditory speech and sign language. Previous studies using these disparate modalities have illustrated similar effects with tight overlap in the pSTS/MTG and the PIFG (MacSweeney 2002; MacSweeney et al. 2006; Spitsyna et al. 2006; Jobard et al. 2007; Lindenberg and Scheef 2007; Pa et al. 2008; Berl et al. 2010; Leonard et al. 2012; Vagharchakian et al. 2012; Regev et al. 2013; Wilson et al. 2018). Therefore we would expect the same production–perception asymmetry across modalities in these regions.

## Conclusion

Our results point towards a possible resolution of a conflict between the functional neuroimaging literature on syntax, which has not identified robust differences between the PIFG and the PTL in syntactic comprehension, and the lesion-symptom mapping literature, which has identified multiple dissociations with respect to damage to these regions. Namely, that activation in language-selective regions of the PTL is driven by the demands of hierarchical structure building necessary to comprehend the meaning of a sentence, whereas activation in language-selective regions of the PIFG is driven by the demands of production, such as converting a structure into a linear string of morphemes. Future neuroimaging studies should seek to provide more direct evidence of a specific functional role for the PIFG in production-related processes.

## Notes

The authors would like to thank an anonymous reviewer, whose suggestions greatly improved the manuscript. *Conflict of Interest*: None declared. 

## Funding

Startup funds at the University of South Carolina (to W.M.); a Magellan Scholar Award from the University of South Carolina (to E.W. and W.M.).

## Supplementary Material

supplementary_6_26_2020_tgaa029
