# Supplementary material for: Syntax-Sensitive Regions of the Posterior Inferior Frontal Gyrus and the Posterior Temporal Lobe Are Differentially Recruited by Production and Perception
Source: Cereb Cortex Commun. 2020 Jul 1;1(1):tgaa029. doi: 10.1093/texcom/tgaa029 (PMC8152856; doi:10.1093/texcom/tgaa029)
Supplement: supplementary_6_26_2020_tgaa029 [file supplementary_6_26_2020_tgaa029.docx]

**Supplementary Materials**

*Degree of overlap of subject-specific activations within the functionally-defined ROIs*

For three out of four of our functionally-defined ROIs, we obtained a comparable degree of overlap of subject-specific activation as Fedorenko et al. (2010); however, for the PIFG ROI defined using the [syntactic > phonological] contrast, we identified 42% less overlap.

Five key differences between the present study and Fedorenko et al. (2010) are: (i) the inclusion of both production and perception (rather than just perception), and (ii) the stimulus design, in which we presented minimal two-word phrases in short blocks of a single trial per block (6s stimulus presentation time) rather than 12-word or 8-word sequences in long stimulus blocks of the same condition in a row (15s or 21s stimulus presentation time) (iii) task, in which half of the data in Fedorenko et al. (2010) had no explicit task, and half the data had a memory probe task, whereas in the present study subjects were required to rehearse the stimulus in the production task and had no explicit task requirement in the perception task, (iv) method of ROI definition: we used the contrast of jabberwocky phrases vs. other conditions to define ROIs, whereas Fedorenko et al. used the contrast of full natural sentences vs. other conditions, and (v) voxel size: 38.44 mm3 in Fedorenko et al., and 15.625 mm3 (acquisition size) and 27 mm3 (reconstructed size) in the present study.

All five of these factors led to a more rigorous ROI definition procedure in our study than in Fedorenko et al. (2010). With respect to (i), as Figure 4 in the main body of the paper shows, in the language-selective subregions of Broca’s area, there was greater activation for phonological production than phonological perception. Thus including production in the definition of the ROIs led to a decreased difference between syntactic and phonological conditions. If our goal is to define language-related ROIs that might potentially subserve both production and comprehension, we believe it is best to include both production and perception rather than using only one task, which may result in a localization bias. With respect to (ii), shorter stimuli reduce the confounds of working memory demands, whether phonological or syntactic working memory (Matchin, 2018), in the perception conditions that are commonly present in longer, more complex structures that likely induce increased activation in Broca’s area (Rogalsky et al., 2008; citation). With respect to (iii), we believe that the absence of a task in half of the data in Fedorenko et al. (2010) reduced the attentiveness of subjects to the meaningless nonword condition, particularly given the particularly long length of blocked stimulus presentation of the same condition: 15s/21s. I.e., subjects likely tuned out to these materials in the scanner, which is consistent with self-report of the subjects in that study (see discussion of this in Fedorenko et al., 2010 – p. 1179, main text and footnotes). Fedorenko et al. (2010) do not report separate data for subjects with and without task, making it difficult to ascertain whether this is the case. While this is also a concern in our study, given that lack of an explicit task in the perception conditions, stimulus length was minimized at three rapid sequences of two nonwords presented simultaneously for a total blocked stimulus presentation of 6s. With respect to (iv), we attempted to define ROIs more specific to syntax by using jabberwocky phrases rather than full real sentences. As shown by Matchin et al. (2017), sentences with all real words produced greater activation in language-related regions than jabberwocky sentences. Fedorenko and colleagues have used full real sentences vs. nonwords to define their ROIs, which we believe conflates lexical, conceptual-semantic, and syntactic processing. Finally, with respect to (v), using smaller voxels results in less ability to detect overlap than bigger voxels. Bigger voxels potentially smooth over smaller subregions, inflating the true degree of overlap relative to smaller voxels.

*Expanded ROI analyses*

The average t-values for each linguistic level (phonological, lexical, syntactic) for both tasks (production & perception) within each ROI is shown in Supplementary Figure 1.


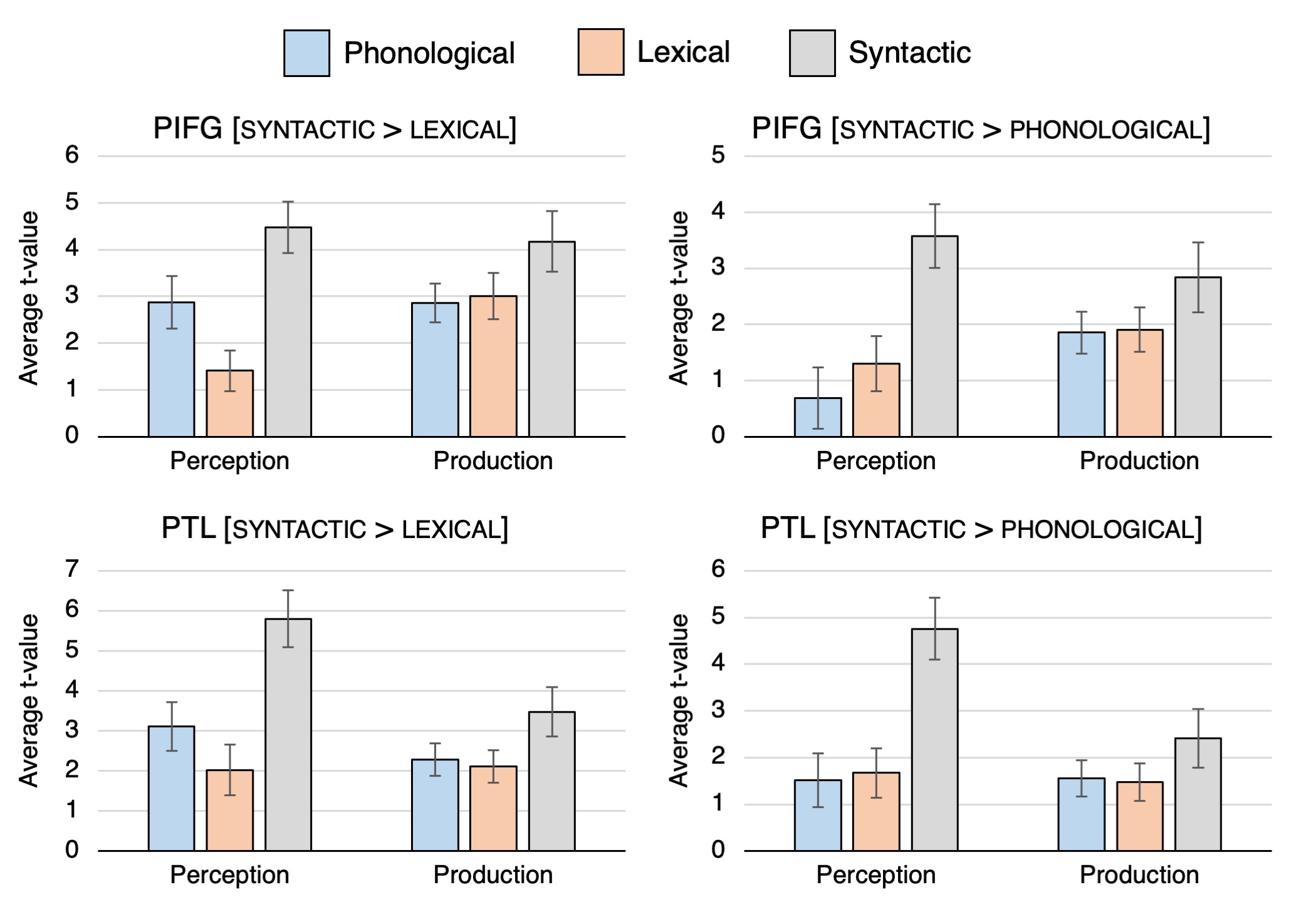


Supplementary Figure 1. Average t-values for each linguistic level (phonological, lexical, syntactic) for both tasks (production & perception) within each ROI. PIFG = posterior inferior frontal gyrus; PTL = posterior temporal lobe. Error bars reflect standard error of the mean.

*Expanded whole-brain analyses*

Individual activation maps associated with the effect of production (perceive+rehearse > perceive+rest) for each linguistic level of Content (phonological, lexical, syntactic) are shown in Supplementary Figure 2, and individual activation maps associated with the effect of perception (continuous perceive > rest) for each linguistic level of Content (phonological, lexical, syntactic) are shown in Supplementary Figure 3. Activations were similar among the levels of content. For the effects of production, all linguistic levels of Content revealed activation in posterior IFG (pars opercularis), precentral gyrus, inferior parietal lobe, posterior STS/MTG, medial occipital cortex, and supplementary motor area (SMA). Activations were bilateral but stronger in the left hemisphere, particularly in frontal and parietal cortex. For the effects of perception, all levels of content revealed activation in left inferior frontal sulcus, left dorsal precentral gyrus, left SMA, and bilateral ventro-lateral occipital-temporal cortex. Additionally, syntactic perception produced significant clusters in left pSTS and right precentral gyrus, and lexical perception produced significant clusters in bilateral basal ganglia and small activations in right precentral gyrus.


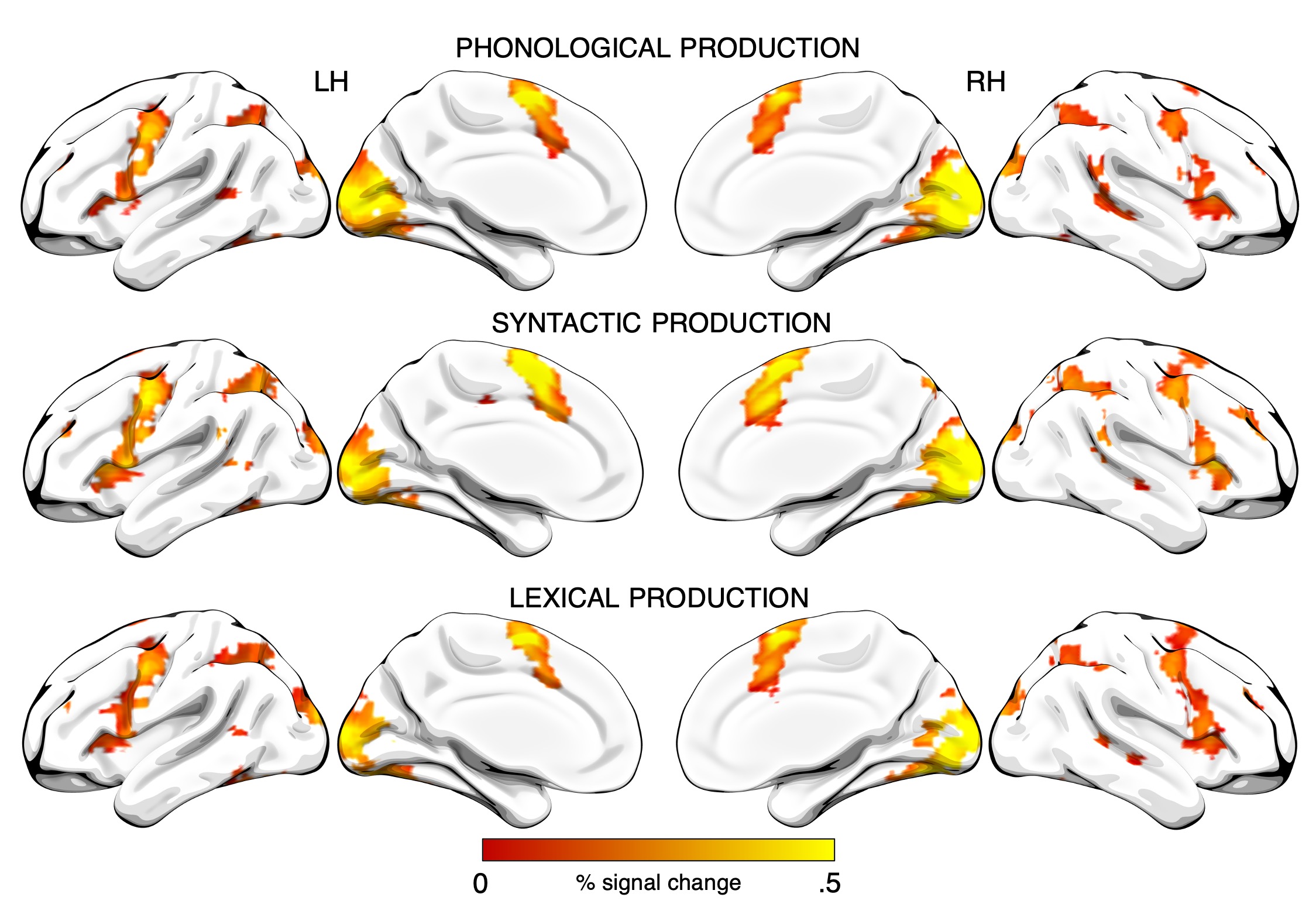


Figure 2. Significant clusters for the effect of production at each linguistic level of Content (phonological, lexical, syntactic) shown on an inflated brain in MNI space.


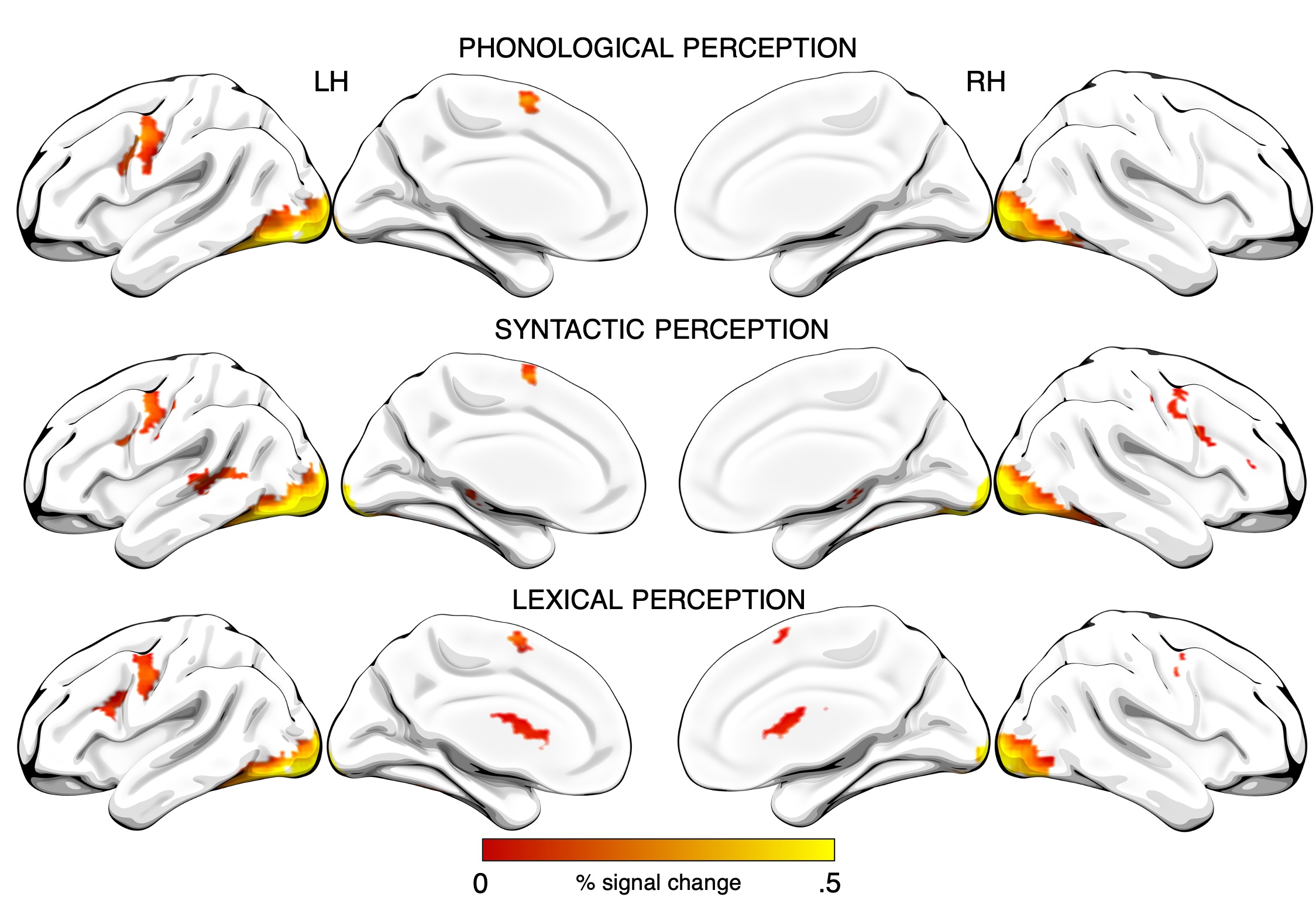


Figure 3. Significant clusters for the effect of perception at each linguistic level of Content (phonological, lexical, syntactic) shown on an inflated brain.

*Full list of materials*

Below is a list of the 64 unique materials for each linguistic level of Content that were used to generate the larger body of stimuli in the experiment.

*Phonological Conditions*

seenig pobset

nillex lerris

ninyo lerris

denferr pobset

seenig furgle

bulbom garlay

tringess ganliff

denferr garlay

denferr lerris

nansow lerris

seenig susset

ninyo demesh

denferr ganliff

nansow pobset

nansow furgle

perwoth ganliff

ninyo furgle

nillex susset

tringess foyrix

seenig lerris

seenig ganliff

nansow demesh

nillex garlay

perwoth susset

bulbom foyrix

bulbom demesh

perwoth furgle

tringess furgle

bulbom lerris

perwoth pobset

nillex furgle

ninyo foyrix

seenig foyrix

tringess garlay

denferr susset

perwoth demesh

nillex foyrix

tringess lerris

bulbom ganliff

nansow ganliff

perwoth foyrix

perwoth garlay

seenig garlay

seenig demesh

denferr foyrix

ninyo pobset

ninyo susset

bulbom furgle

nansow garlay

nillex ganliff

nillex demesh

denferr furgle

bulbom susset

denferr demesh

bulbom pobset

tringess demesh

perwoth lerris

ninyo ganliff

nillex pobset

ninyo garlay

nansow foyrix

tringess susset

tringess pobset

nansow susset

*Lexical Conditions*

pirate garbage

mutant pistol

mutant garbage

hermit pistol

glutton dogma

sheriff organ

glutton vodka

sheriff robot

glutton robot

pirate scandal

poet scandal

sheriff pistol

mutant organ

poet organ

mutant scandal

hermit robot

sheriff dogma

ninja organ

hostage pistol

mutant robot

ninja fortress

hermit organ

pirate robot

hostage vodka

mutant vodka

ninja vodka

hostage robot

ninja garbage

pirate organ

mutant dogma

pirate dogma

hermit vodka

ninja scandal

poet robot

pirate fortress

sheriff vodka

mutant fortress

glutton pistol

hostage fortress

sheriff garbage

hermit fortress

hermit dogma

hermit scandal

poet pistol

sheriff fortress

sheriff scandal

hostage garbage

hostage organ

pirate pistol

glutton organ

hostage scandal

glutton scandal

poet garbage

pirate vodka

ninja robot

glutton garbage

ninja dogma

hermit garbage

poet dogma

poet vodka

ninja pistol

glutton fortress

poet fortress

hostage dogma

*Syntactic Conditions*

few sheeves those bleffs

his larts the sheeves

each pand a clope

the delks his pands

his glits few pands

each woon those sheeves

their woon each glit

a glit the bleffs

his delks their bleff

the pands his bleffs

a sheeve the woons

this woon those pands

his pands each woon

this lart their glit

few woons those clopes

this sheeve a woon

few glits the larts

few pands those larts

a pand those delks

few clopes those glits

this clope his sheeves

their delk his glits

each glit those woons

his sheeves this glit

his bleffs the clopes

those clopes few larts

their bleff the delks

their clope this pand

the clopes their lart

this bleff each delk

a clope this bleff

a lart this clope

a woon each pand

the woons each lart

those delks their sheeve

their pand his delks

each clope their delk

a delk his clopes

each delk their pand

each sheeve a pand

this pand their woon

those sheeves his woons

those larts each clope

his clopes this woon

the bleffs this lart

each bleff a lart

a bleff this delk

those bleffs the glits

their glit few bleffs

the glits each bleff

those pands few delks

his woons each sheeve

the larts a delk

their sheeve a bleff

few bleffs a sheeve

those glits few sheeves

few larts this sheeve

this delk their clope

this glit the pands

their lart a glit

the sheeves few woons

few delks his larts

each lart few glits

those woons few clopes
